# Factors Influencing Retention Intention of Nurses at Long-term Care Hospitals in Korea

**DOI:** 10.1093/geroni/igab046.3192

**Published:** 2021-12-17

**Authors:** So Young Shin, Joo Hee Kim

**Affiliations:** 1 Global Brain Health Institute, University of California, San Francisco / Inje University, Busan, Pusan-jikhalsi, Republic of Korea; 2 Busan Hangun Hospital, Busan, Pusan-jikhalsi, Republic of Korea

## Abstract

Purpose: This study aimed to investigate the levels and correlations of role conflict, nursing professionalism, nursing work environment and retention intention, and the factors influencing retention intention of nurses at long-term care hospitals. Methods: The subjects included 183 nurses at nine long-term care hospitals in one metropolitan city in Korea. A set of self-reported questionnaires was administered to assess general characteristics, role conflict, nursing professionalism, nursing work environment, and retention intention of the subjects. Collected data was analyzed using descriptive statistics, t-tests, one-way ANOVA, Pearson correlation coefficients, and multiple linear regression. Results: 183 subjects with a mean (±SD) age of 41.66 (±12.29) years were included in the final analyses. Retention intention had a significant positive correlation with nursing professionalism (r=.39, p<.001) and nursing work environment (r=.51, p<.001). Nursing work environment had a significant negative correlation with role conflict (r=-.30, p<.001) and a significant positive correlation with nursing professionalism (r=.48, p<.001). In the final multiple regression analysis, the factors influencing retention intention of subjects were number of beds (β=-.15 p<.026), nursing professionalism (β=.19, p=.007) and nursing work environment (β=.36, p<.001). The explanatory power of number of beds, nursing professionalism and nursing work environment on retention intention was 34.0% (F=16.66, p<.001). Conclusion: Improving nursing professionalism and nursing work environment of nurses at long-term care hospitals will ultimately enhance their retention intention and positively impact on the quality of gerontological nursing service.

